# Cognitive, emotional, physical, and behavioral stress-related symptoms and coping strategies among university students during the third wave of COVID-19 pandemic

**DOI:** 10.3389/fpsyt.2022.933981

**Published:** 2022-09-16

**Authors:** Merna Attia, Fatma A. Ibrahim, Mohamed Abd-Elfatah Elsady, Mohamed Khaled Khorkhash, Marwa Abdelazim Rizk, Jaffer Shah, Samar A. Amer

**Affiliations:** ^1^Faculty of Medicine, Zagazig University, Zagazig, Egypt; ^2^New York State Department of Health, New York, NY, United States; ^3^Department of Public Health and Community Medicine, Faculty of Medicine, Zagazig University, Zagazig, Egypt; ^4^Member at Royal Colleague of General Practitioners [INT], London, United Kingdom; ^5^Department of Mental Health Primary Care, Nova University, Lisbon, Portugal

**Keywords:** stress, stress-related symptoms, coping strategies, university students, the COVID-19 pandemic, Egypt

## Abstract

**Background:**

Stress is manifested by different physical, cognitive, emotional, and behavioral stress-related symptoms, and everyone experiences it uniquely. The COVID-19 Pandemic has tremendously affected university students' lives. So, we conducted this study to determine the stress frequency, causes, determinants, and related symptoms involving physical, cognitive, emotional, and behavioral traits and coping strategies among university students in Egypt during the third wave of the COVID-19 pandemic, 2021.

**Methods:**

Cross-sectional study targeted 1,467 randomly selected undergraduate university students, representing all colleges from 30 universities in Egypt, through a validated self-administrated questionnaire.

**Results:**

The total stress-related symptom score was statistically significant (*p* < 0.05), higher among females, married, living on campus, with a (B) GPA, and those who had both organic and psychological disorders. The top 10 prevalent physical symptoms were headaches, chronic fatigue, hair loss, low back pain, neck pain, shoulders and arm pain, ophthalmological symptoms, acne, shakiness of extremities, and palpitations, respectively. The most reported symptoms regarding the cognitive, emotional, and behavioral aspects were anxiety and racing thoughts, moodiness and irritability, and excessive sleeping, respectively. Nine hundred and thirty-seven (63.9%) reported that the COVID-19 pandemic badly affected their lives, either directly or indirectly. The study showed that the prevalence of stress among university students is more than 97%. One thousand and five (68.5%) preferred isolation as a relieving technique.

**Conclusion:**

Stress and its related physical, cognitive, emotional, and behavioral symptoms are prevalent among university students. Most of the university students who were recruited reported that the COVID-19 pandemic badly affected their lives and used negative ways to deal with stress, like staying alone and sleeping too much. Positive ways to deal with stress, like seeing a therapist or meditating, were less common.

## Introduction

Stress is the physiologic response in which various defensive mechanisms come into play to deal with a threatening or challenging situation (1). During stress, the nervous system releases stress hormones that trigger a fight or flight state. Stress can be positive, as the stress response helps people stay alert when needed. After the stressors have passed, the body returns to normal and relaxes. But experiencing stress frequently or constantly can cause wear and tear on different body systems. So, both mental and physical health can be badly affected (2).

Stress and its related symptoms can be triggered or exaggerated by many risk factors, including age, gender, marital status, personal characteristics, grade level, academic performance (3, 4), nature of the study, living conditions, financial problems (5), work environment (6), quality of sleep (7), chronic illness (8), facing massive life changes (9), and facing a pandemic like COVID-19 (10).

Worldwide, academic stress is very prevalent among university students, negatively affecting their mental health (11–14). Medical students are the most affected by stress and depression (15–17). Academic stress occurs due to the requirements of college life, such as frequent examinations, deadlines, poor time management, delayed assignments, poor housing, noise, overcrowding, working while studying, adjustment to a new environment or even country, the imbalance between academic and personal life, and many other personal problems (18).

The COVID-19 Pandemic has tremendously affected people's lives. University students were also affected physically and psychologically (19). Recognizably, college students had a higher prevalence of stress and other mental health issues during the pandemic (10). Many risk factors are implicated in increasing perceived stress during the pandemic, such as quarantine for more than 14 days, inadequate access to supplies, having a chronic medical illness, fear of infection, getting infected, or the death of a relative or loved one (20–22).

Stress is manifested by different symptoms, as everyone experiences stress uniquely. It may involve physical, cognitive, emotional, and behavioral symptoms (23, 24). Physical symptoms include musculoskeletal pain, chronic fatigue, breathing problems, palpitations, bloating, abdominal pain, and changes in bowel habits (25). Some other physical symptoms include headaches and migraines (23) and hair loss (26), as well as acne (27–29) and eye problems (30, 31).

Cognitive aspects include the following symptoms: poor concentration, memory problems, constant worrying and anxiety, and seeing the negatives only. Emotional symptoms include depression, agitation, irritability, and loneliness. Behavioral symptoms include: neglecting responsibilities, appetite changes, sleep disturbances, smoking, using alcohol or drugs, and various nervous acts such as nail biting (23).

Ignoring ongoing, chronic stress and its related symptoms can cause or worsen many serious health problems, including mental health problems, such as depression, anxiety, and personality disorders, which would reflect on their social and academic lives. It can also cause cardiovascular diseases, including heart disease, high blood pressure, abnormal heart rhythms, heart attacks, and strokes; obesity and other eating disorders; menstrual problems; sexual dysfunction, such as impotence and premature ejaculation in men and loss of sexual desire in men and women; skin and hair problems, such as acne, psoriasis, and eczema; and permanent hair loss; and gastrointestinal problems, such as GERD, gastritis, ulcerative colitis, and irritable colon (32).

People face stress with various coping strategies to reduce the pressure of stress. These strategies can be positive or negative. Positive techniques include: exercise as a positive strategy to divert attention from unfavorable thoughts and stop the stress cycle; hormones that reduce stress can be released while you are in a face-to-face interaction with a friend or relative; By triggering the body's relaxation response, relaxation techniques lower tension and increase emotions of joy; Getting enough sleep helps with fatigue relief and stress reduction; eating a healthy diet rich in fresh fruit and vegetables, omega-3 fatty acids, and high-quality protein helps to cope better with ups and downs (23, 33); and A psychiatric consultation can help you customize the most effective stress management techniques for your needs and lifestyle.

Negative stress-coping techniques include social isolation and loneliness that raise health risks comparable to those associated with alcohol use disorders or smoking 15 cigarettes a day, increasing the risk of premature mortality. Additionally, they are twice as detrimental to both physical and mental health as obesity (34, 35). Some people might believe that smoking or alcohol temporarily lessens stress. But over time, they can exacerbate stress, increasing the likelihood of mental and psychological issues in addition to alcohol dependence (36). Overeating processed and convenience foods, refined carbs, and sugary snacks can exacerbate the effects of stress on the body and have a negative impact (23).

Worldwide, COVID-19's spread is uncontrollable, and it has already had an impact on people and countries. Holmes et al. called for multidisciplinary scientific research to be at the forefront of the international response to the COVID-19 pandemic in order to provide evidence-based guidance on how to promote people's health and well-being during the pandemic (37). In response to this call, we're focusing on university students as a vulnerable group to age-related, academic, and COVID-19 related stressors during the third wave of the COVID-19 pandemic to learn more about their stress and health problems. So, we conducted this study to improve the overall health status and reduce the impact of stress among university students through determining and studying the stress prevalence, causes, determinants, stress-related symptoms (physical, cognitive, emotional, and behavioral symptoms) and stress coping strategies among undergraduate university students in Egypt during the COVID-19 pandemic, 2021.

## Methods

### Study design, and participants

The cross-section study targeted randomly selected undergraduate university students in Egypt who fulfilled the selection criteria that included undergraduate students, aged between 17 and 26, from public and private universities in Egypt and resident in Egypt during the study period, excluding students who had chronically complicated medical or psychological disorders that interfered with their participation such as complicated diabetes (Retinal detachment, diabetic foot, diabetic nephropathy), and complicated cardiovascular diseases e.g., chronic heart failure.

### Sample size and sample techniques

Egypt currently has 20 public universities (with about two million students) and 23 private universities (about 60,000 students). The sample size was calculated to be 489 by using the Epi–info program, which used the total number of 2,442,000 university students in Egypt, and due to a lack of data on the frequency of stress-related symptoms, we assumed that it was 50% based on the average prevalence of perceived stress scale in other studies in Egypt [62.3% in Fayoum, in 2017, and 54% in Ain Shams University in 2016] (38). As a multistage cluster sample, after considering the effect design, the sample size was tripled to increase the power of the study and to represent all targeted strata, which totaled 1,467 students.

A multistage sampling technique was used to recruit a representative sample through the following stages: (1) A random selection of two or three faculties from the 20 Egyptian governorates that contain universities, with the exception of Cairo (Egypt's capital, which contains more than 260,000 students and more than 43 faculties, and home to the majority of private universities in Egypt), from which we target 10 universities. (2) The sample was stratified by college or faculty type (13 practical and 13 theoretical) within each college. (3) The sample is then stratified by grade level for each college. (4) A simple random sample obtained from each university's most popular official social media platforms or websites for these colleges and classes (Facebook, WhatsApp, and Telegram groups). Students completed and submitted the self-administered online questionnaire after providing informed written consent. Reminder messages and reposting of the questionnaire's link were used to increase the response rate until the required sample is completed (see Supplementary materials 1, 2).

### Data collection tool

The Google form of the Arabic questionnaire, as it is the main language in Egypt, was designed to achieve the objectives of the study based on previous studies (23, 25–31, 33, 39), and was prepared as a well-structured, pretested, pre-coded, Arabic, self-administrated questionnaire. It was originally created in English before being translated into Arabic, except Section 4 (the Perceived Stress Scale), which had an Arabic validation in another study (39). The English version of the questionnaire was translated into Arabic by a bilingual panel consisting of two healthcare professionals and one externally qualified medical translator. Two translators who spoke English did a back translation, and if there were any questions, the original panel was asked.

A validating schedule was developed by six experts (three psychiatrists, one family medicine, and two public health and community medicine) for the questionnaire, after which it was tested for clarity and comprehension through a pilot study, which was conducted on 25 students. Their results were included in our study results. The calculated Cronbach's alpha was 0.79. It is composed of five main sections (see Supplementary material 3).

#### Section 1: Sociodemographic data

There were 11 questions (Q) that asked about their gender, age, nationality, marital status, residence, university name, college name, study nature, academic level, GPA, and if they had any health problems.

#### **Section 2**: Stress-related symptoms

It is composed of 54 questions, that were well structured from the following studies (23, 25–31) to assess the four main domains (symptoms);

- Physical symptoms are assessed through 36 questions to evaluate the affection of eight systems as follows: the neuro-musculoskeletal system (5 Q). The gastrointestinal system (10 Q). The cardiovascular system (3 Q). Cutaneous system (skin and hair symptoms) (3 Q). The respiratory system (2 Q). The ophthalmological system (1 Q). The gynecological system (1 Q) (See Section 2 of the questionnaire).
- Cognitive symptoms are assessed through five Qs about concentration, memory problems, anxiety and overthinking, pessimism, and decision-making.
- Emotional symptoms are assessed through five Qs about depression and unhappiness, anxiety and agitation, feeling loneliness and isolation, moodiness, irritability, and anger, and if there are any other mental or emotional health problems.
- Behavioral symptoms are measured by answering eight questions about appetite, excessive sleeping, insomnia, withdrawing from groups and preferring to be alone, not doing their jobs, smoking, and biting their nails.

##### The stress-related symptoms scaling method

- Each question measured the occurrence rate of each symptom on the Likert Scale.
- The Likert scale has four points (never = zero, rarely = one, frequently or occasionally = two, and always = three).
- The total score of stress-related symptoms was 162; the physical symptom score was 108, the cognitive symptom score was 15, the emotional score was 15, and the behavioral score was 24.
- The top 10 symptoms were calculated by the sum of (often, sometimes, and always).

#### Section 3: About the COVID-19 pandemic (exposure history, effect on the study)

Exposure or infection history (themselves or their families), and students were allowed to select more than one answer (See Section 3 of the questionnaire).

Studying effects, such as isolation at home with suspension of study for a period of time, studying online, delayed exams, and exams replaced with research. All these options were introduced to participants as Egypt considered all these options as mitigation measures for students during the pandemic, and they differed according to the college and the nature of the study.

#### Section 4: Perceived stress scale (PSS-10)

It's a 10-question validated scale (with the internal consistency of the PSS-10 and its positive and negative subscales were acceptable (coefficient alpha = 0.67, 0.79, and 0.89), to assess the severity of the perceived stress on a 4-point Likert Scale (never = zero, almost never = one, sometimes = two, often = three, and very often = four). Based on the PSS-10, the stress scale was classified into three categories: (0–13) low perceived stress, (14–26) moderate perceived stress, and (27–40) high-perceived stress (39).

#### Section 5: Stress-relieving techniques

University students were allowed to select more than one answer from many suggested coping strategies, such as isolation, sleeping, eating, praying, etc., and others, and write down their other coping strategies if they had them (see Section 5 of the questionnaire below). From articles by the Anxiety and Depression Association of America (ADAA) and the CDC, we came up with these choices (40, 41).

### Ethical consideration, and method of data collection

After the Exempt No. 6925-14-7-2021, IRP ethical approval was obtained from the institutional review board at Zagazig University, Egypt. The official college classrooms' online social media portals, such as Facebook, WhatsApp, and Telegram, were used to distribute the online Google form. The data was collected during May, 2021. To increase the response rate, we asked the admins to share, repost, and invite them to do so after they signed a written agreement that their identities would not be revealed and a weekly reminder until they finished the sample.

### Data analysis

The collected data was coded and analyzed using the SPSS program version 23.0. The categorical variables were expressed as frequency (F) and percentage (%), and the chi-square test (*X*^2^) was used for analysis. Quantitative variables were presented as mean ± standard deviation (SD) or median according to the data distribution. The Kolmogorov-Smirnov test was used to determine whether continuous data was considered skewed or not normally distributed. A *t*-test was used to confirm this. The normally distributed variables were analyzed using ANOVA and Post-Hoc tests, while non-parametric variables were analyzed using the Mann-Whitey U test, with statistical significance set at *p* 0.05. To test the association between two continuous variables persons correlation coefficient test was used. A simple logistic regression analysis within A generalized linear model technique was used to examine the association of each potential factor. With the binary outcome of vaccination adverse effects (no or mild stress, and moderate or severe stress).

## Results

### Socio-demographics of the university students

Out of the 1,467 students, 1,157 (78.9%) were females, with a mean age of 23.0 years (SD = 21.2 ± 1.7), 1,431 (97.6%) were Egyptians, 1,262 (86.0%) were single, 653 (44.5%) living in urban areas, and 991 (76.6%) in practical colleges, with a (B) GPA of 593 (40.4%), and 761 (51.9%) were without comorbidities (Table 1).

**Table 1:** The Sociodemographic Characteristics, and its relationship with the stress-related symptoms score (per domains and total score).

**Sociodemographic variables**	**Total sample *F* (%)**	**The main domains of stress-related symptoms scores**				**The total score of stress symptoms Mean ±SD**
		**Physical symptoms Mean ±SD**	**Behavioral symptoms Mean ±SD**	**Cognitive symptoms Mean ±SD**	**Emotional symptoms Mean ±SD**	
**Sex**						
Female	1,157 (78.9)	52.9 ± 17.8	12.4 ± 3.4	10.7 ± 3.0	10.4 ± 3.3	86.4 ± 24.0
Male	310 (21.1)	38.8 ± 19.7	12.1 ± 4.5	9.8 ± 3.7	9.6 ± 3.9	70.2 ± 28.3
*P*-values of student *t*-test		0.00^*^	0.24	0.00^*^	0.00^*^	0.00^*^
**Nationality**						
Non-Egyptian	35 (2.4)	42 (44.3 ± 19.9)	12 ± 4.7	9.9 ± 3.1	9.6 ± 4.0	75.8 ± 28.4
Egyptian	1,431 (97.6)	50 (49.3 ± 18.9)	12.3 ± 3.6	10.5 ± 3.2	10.2 ± 3.5	82.4 ± 25.9
P values of student *t*-test		0.07^##^	0.19	0.17	0.34	0.07
**College**						
Practical	991 (76.6)	49 (49.5 ± 18.9)	12.3 ± 3.5	10.7 ± 3.1	10.4 ± 3.4	82.8 ± 25.4
Theoretical	302 (23.4)	50 (48.3 ± 19.1)	12.3 ± 4.0	10.2 ± 3.5	9.8 ± 3.8	80.6 ± 26.8
P values of student *t*-test		0.08^##^	0.33	0.21	0.11	0.07
**Marital status**						
Single	1,262 (86.0)	49 (48.7 ± 19.0)	12.3 ± 3.6	10.5 ± 3.2	10.2 ± 3.5	81.7 ± 25.9
In relationship	53 (3.7)	50 (50.7 ± 19.7)	12.6 ± 3.5	10.6 ± 2.9	11.0 ± 2.9	84.9 ± 25.6
Married	43 (2.9)	49 (52 ± 19.7)	13.2 ± 5.0	10.7 ± 3.9	10.3 ± 4.2	86.2 ± 29.9
Engaged	108 (7.4)	52 (52.5 ± 17.3)	12.3 ± 3.1	10.7 ± 3.1	10.1 ± 3.5	85.6 ± 23.7
P of F test		0.03^*^^#^	0.11	0.29	0.06	0.02^*^
**Residence**						
Campus	216 (14.7)	51 (52.2 ± 18.7)	12.6 ± 3.4	10.9 ± 2.9	10.7 ± 3.3	86.4 ± 24.7
Urban	653 (44.5)	48 (47.7 ± 18.6)	12.2 ± 3.7	10.5 ± 3.2	10.2 ± 3.5	80.6 ± 25.3
Rural	598 (40.8)	51 (49.7 ± 19.3)	12.3 ± 3.7	10.4 ± 3.3	10.1 ± 3.5	82.5 ± 26.5
P of F test		0.04^*^^#^	0.68	0.71	0.33	0.00^*^
**GPA**						
Excellent (A)	312 (21.3)	46 (46.3 ± 18.8)	11.6 ± 3.5	9.9 ± 3.1	10.0 ± 3.6	77.9 ± 25.5
Very good (B)	593 (40.4)	51 (50.6 ± 19.4)	12.4 ± 3.3	10.7 ± 3.0	10.3 ± 3.3	84.3 ± 25.1
Good (C)	417 (28.7)	51 (49.9 ± 18.6)	12.6 ± 3.8	10.6 ± 3.3	10.4 ± 3.5	83.5 ± 25.9
Fairly good (D)	145 (9.9)	46 (46.1 ± 18.9)	12.6 ± 4.5	10.9 ± 3.6	10.1 ± 4.1	79.6 ± 27.9
P of F test		0.00^*^^#^	0.02^*^	0.001^*^	0.39	0.00^*^
**Had chronic diseases**						
Non	761 (51.9)	42 (42.9 ± 18.3)	11.4 ± 3.7	9.7 ± 3.3	9.1 ± 3.6	73.1 ± 25.2
Organic	105 (7.2)	53 (52.8 ± 16.2)	11.8 ± 3.3	9.1 ± 2.9	9.1 ± 3.1	82.9 ± 22.7
Psychological	294 (20.2)	52 (51.3 ± 16.3)	13.2 ± 3.3	11.6 ± 2.7	11.6 ± 2.8	87.7 ± 21.3
Both	307 (20.9)	63 (61.5 ± 17.1)	13.7 ± 3.2	12.1 ± 2.3	12.1 ± 2.7	99.5 ± 21.5
P of F test		0.00^*^^#^	0.00^*^	0.00^*^	0.00^*^	0.00^*^

* p < 0.05–there is a statistically significant relationship/all p-values are to.

# P for Kruskall Wallis test.

## p-values of MannWhitney U tset.

### Stress-related symptoms

**Figure 1:**
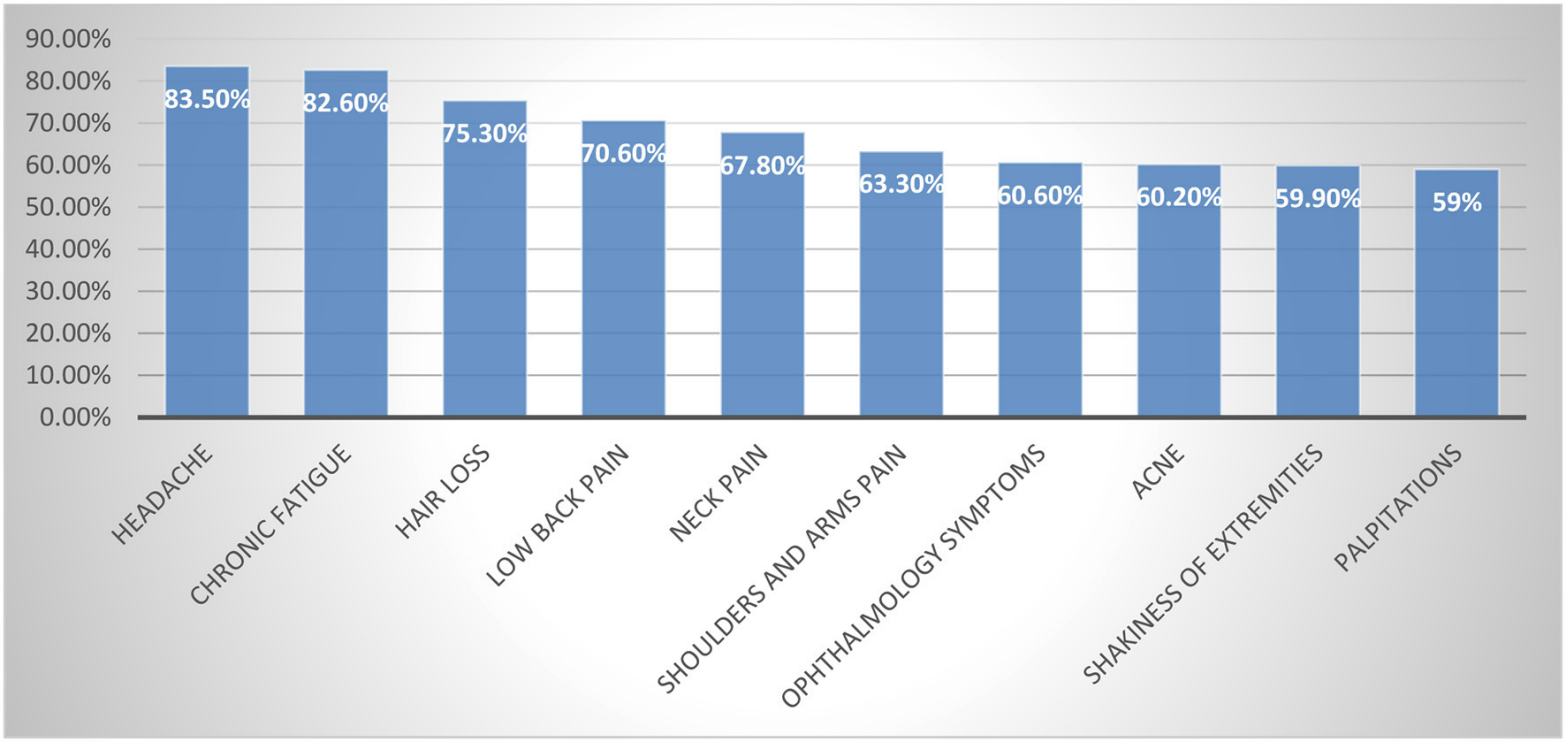
The frequency of the top 10 stress-related physical symptoms among the studied university students. Top ten symptoms were calculated by the sum of (often or sometimes and always).

**As regards the stress-related physical symptoms**, the top 10 symptoms in descending order were: headache 1,225 (83.5%), chronic fatigue 1,212 (82.6%), hair loss 1,104 (75.3%), low back pain 1,036 (70.6%), neck pain 994 (67.8%), shoulders and arms pain 929 (63.3%), ophthalmology related symptoms 889 (60.6%), acne 883 (60.2%), shakiness of extremities such as hands 879 (59.9%), and palpitations 866 (59%). On the other hand, the following symptoms were experienced as never before: difficulty swallowing, painful oral ulcers, and increased body temperature, by 695 (47.4%), 664 (45.3%), and 594 (40.5%), respectively (Table 2; Figure 1).

**Table 2:** Stress-related Physical symptoms among university students.

**Physical symptoms**	**Never *F* (%)**	**Rare** ***F* (%)**	**Often or sometimes** ***F* (%)**	**Always** ***F* (%)**	Sum^**^F (%)	**Total score**
**Neuro-musculoskeletal system**						
Back pain	168 (11.5)	263 (17.9)	684 (46.6)	352 (24.0)	1,036 (70.6)	Mean ± SD (range)
Neck pain	180 (12.3)	293 (19.9)	661 (45.1)	333 (22.7)	994 (67.8)	
Shoulders and arms pain	200 (13.6)	338 (23.0)	610 (41.6)	319 (21.7)	929 (63.3)	
Shakiness of extremities such as hands	235 (16.0)	353 (24.1)	536 (36.5)	343 (23.4)	879 (59.9)	
Dizziness	228 (15.5)	386 (26.3)	582 (39.7)	271 (18.5)	853 (58.2)	
**General symptoms (Nonspecific systems)**						
Clenching	492 (33.5)	400 (27.3)	355 (24.2)	220 (15.0)	575 (39.2)	49.2 ± 18.9 (0-108)
Headache	61 (4.2)	181 (12.3)	635 (43.3)	590 (40.2)	1,225 (83.5)	
Migraine	264 (18.0)	342 (23.3)	516 (35.2)	345 (23.5)	861 (58.7)	
Chronic fatigue	76 (5.2)	179 (12.2)	510 (34.8)	702 (47.9)	1,212 (82.6)	
Increased temperature	494 (33.7)	647 (44.1)	274 (18.7)	52 (3.5)	326 (22.2)	
Hotness or redness in face, ears, neck or chest (Hot flashes)	504 (34.4)	485 (33.1)	350 (23.9)	128 (8.7)	478 (32.6)	
Increased sweating	370 (25.2)	450 (30.7)	423 (28.8)	224 (15.3)	647 (44.1)	
Slurred speech	435 (29.7)	404 (27.5)	428 (29.2)	200 (13.6)	628 (42.8)	
Frequent cold or flu	370 (25.2)	539 (36.7)	383 (26.1)	175 (11.9)	558 (38)	
Increased weight	697 (47.5)	273 (18.0)	295 (20.1)	202 (13.8)	497 (33.9)	
Decreased weight	535 (36.5)	343 (23.4)	310 (21.1)	279 (19.0)	589 (40.1)	
**Gastro intestinal**						
Dry mouth	360 (24.5)	475 (32.4)	446 (30.4)	186 (12.7)	632 (43)	
Painful oral ulcers	664 (45.3)	433 (29.5)	253 (17.2)	117 (8.0)	370 (25.2)	
Difficult swallowing	695 (47.4)	477 (32.5)	228 (15.5)	67 (4.6)	295 (20.1)	
Heartburn or regurgitation after eating	414 (28.2)	396 (27.0)	416 (28.4)	241 (16.4)	657 (44.8)	
Vomiting	466 (31.8)	474 (32.3)	337 (23.0)	190 (13.0)	527 (36)	
Stomachache after eating	377 (25.7)	409 (27.9)	420 (28.6)	261 (17.8)	681 (46.4)	
Abdominal pain	250 (17.0)	395 (26.9)	543 (37.0)	279 (19.0)	822 (56)	
Bloating sensation and abdominal discomfort	271 (18.5)	335 (22.8)	534 (36.4)	327 (22.3)	861 (58.7)	
Diarrhea	414 (28.2)	641 (43.7)	315 (21.5)	97 (6.6)	412 (28)	
Constipation	364 (24.8)	536 (36.5)	394 (26.9)	173 (11.8)	567 (38.7)	
**Respiratory system**						
Rapid breathing	437 (29.8)	449 (30.6)	409 (27.9)	172 (11.7)	581 (39.6)	
Shortness of breath	352 (24.0)	447 (30.5)	435 (29.7)	233 (15.9)	668 (45.5)	
**Cardio vascular system**						
Chest pain	482 (32.9)	453 (30.9)	371 (25.3)	161 (11.0)	532 (36.3)	
Palpitations	211 (14.4)	390 (26.6)	575 (39.2)	291 (19.8)	866 (59)	
Increased blood pressure	594 (40.5)	449 (30.6)	311 (21.2)	113 (7.7)	424 (28.9)	
**Dermatology system**						
Acne	277 (18.9)	307 (20.9)	390 (26.6)	493 (33.6)	883 (60.2)	
Hair loss	168 (11.5)	195 (13.3)	414 (28.2)	690 (47.0)	1,104 (75.3)	
Itching	434 (29.6)	462 (31.5)	362 (24.7)	209 (14.2)	571 (38.9)	
**Ophthalmology**						
Blurred vision, double vision, fogginess, and any other eye-related complaints	259 (17.7)	319 (21.7)	483 (32.9)	406 (27.7)	889 (60.6)	
**Female menstrual disturbance (*****T*** **= 1,157)**						
Menstrual disturbance	179 (15.5)	311 (26.9)	361 (31.2)	306 (26.4)	667 (57.6)	

** Sum of (always +often or sometimes).

**As regards cognitive, emotional, and behavioral stress-related symptoms**, university students reported that the most common cognitive symptoms were anxiety and racing thoughts 1,313 (89.5%), followed by poor concentration 1,274 (86.8%). Emotional symptoms including moodiness, irritability, or anger were 1,288 (87.8%), and depression and unhappiness were 1,232 (84%). Behavioral symptoms were excessive sleeping 1,142 (77.8%), followed by avoiding gatherings and preferring isolation 1,131 (77%) (Table 3).

**Table 3:** Cognitive, emotional and behavioral stress-related symptoms among students.

	**Never** ***F* (%)**	**Rare** ***F* (%)**	**Often or sometimes** ***F* (%)**	**Always** ***F* (%)**	^**^Sum ***F* (%)**	**Total score Mean ±SD (range)**
**Cognitive symptoms**						
Poor concentration	43 (2.9)	150 (10.2)	800 (54.5)	474 (32.3)	1,274 (86.8)	Cognitive =10.5 ± 3.1 (0–15)
Memory problems	52 (3.5)	165 (11.2)	704 (48.0)	546 (37.5)	1,250 (85.2)	
Anxiety or Racing thoughts	34 (2.3)	120 (8.2)	470 (32.0)	843 (57.5)	1,313 (89.5)	
Seeing only the negative	154 (10.5)	386 (26.3)	504 (34.4)	423 (28.8)	927 (63.2)	
Inability to take proper decisions (poor judgment)	107 (7.3)	348 (23.7)	583 (39.7)	429 (29.2)	1,012 (69)	
**Emotional symptoms**						
Depression and unhappiness	58 (4.0)	177 (12.1)	639 (43.6)	593 (40.4)	1,232 (84)	Emotional = 10.2 ± 3.5 (0–15)
Anxiety and agitation	97 (6.6)	207 (14.1)	613 (41.8)	550 (37.5)	1,163 (79.3)	
Feeling of loneliness and isolation	81 (5.5)	185 (12.6)	501 (34.2)	700 (47.7)	1,201 (81.9)	
Moodiness, irritability or anger	50 (3.4)	129 (8.8)	543 (37.0)	745 (50.8)	1,288 (87.8)	
Other mental or emotional health problems	462 (31.5)	355 (24.2)	351 (23.9)	299 (20.4)	650 (44.3)	
**Behavioral symptoms**						
Increased appetite	362 (24.7)	380 (25.9)	496 (33.8)	229 (15.6)	725 (49.4)	Behavioral = 12.3 ± 3.6 (0–24)
Decreased appetite	257 (17.5)	356 (24.3)	543 (37.0)	311 (21.2)	854 (58.2)	
Excessive sleeping	106 (7.2)	219 (14.9)	518 (35.5)	624 (42.5)	1,142 (77.8)	
Insomnia	182 (12.4)	353 (24.1)	565 (38.5)	367 (25.0)	932 (63.5)	
Withdrawing from gatherings	118 (8.0)	218 (14.9)	450 (30.7)	681 (46.4)	1,131 (77)	
Neglecting responsibilities	133 (9.1)	322 (21.9)	539 (36.7)	473 (32.2)	1,012 (69)	
Smoking	1,315 (89.6)	65 (4.4)	30 (2.0)	57 (3.9)	87 (5.9)	
Nail biting	682 (46.5)	242 (16.5)	248 (16.9)	295 (20.1)	543 (37)	

** Sum of (always +often or sometimes).

**The stress-related symptom score:** The mean score of total stress-related symptoms was significantly (*p* < 0.05) higher in females (86.4 ± 24.0), married (86.2 ± 29.9), living on campus (86.4 ± 24.7), B GPA (84.3 ± 25.1), and students who had both organic and psychological disorders (99.5 ± 21.5). The total number of stress-related symptoms had no statistically significant (*p* > 0.05) relationship with either nationality or faculty type (Table 1).

### Determinants of the stress related symptoms

Stress-related symptoms were significantly higher among females (*p* = 0.001). Cognitive and emotional symptoms were significantly higher among females. Stress symptoms were significantly associated with stressed married students (*p* = 0.02), particularly the physical symptoms (*p* = 0.03). They were significantly higher among students living on campus (*p* = 0.00), particularly physical symptoms (*p* = 0.04). Students with a B GPA showed a significant prevalence of stress symptoms (*p* = 0.001), particularly physical (*p* = 0.001), cognitive (*p* = 0.001), and behavioral (*p* = 0.02). Students who suffered from organic and psychological disorders revealed significantly prevalent stress symptoms involving the four domains (*p* = 0.001).

### Effect of the COVID-19 pandemic on university students

Nine hundred thirty-seven university students (63.9%) reported that COVID-19 affected their lives directly and/or indirectly. As regards the exposure/infection history, 708 (72.8%) got mild COVID-19 symptoms, 842 (57.4%) were isolated at home, and 377 (38.5%) had one of their relatives die after infection. As regards the effect of the COVID-19 Pandemic on their studies, 783 (53.4%) studied online, and 664 (45.3%) of their exams were delayed. There was a moderate to good (*r* > 0.76) positive or direct statistically significant association (*p* = 0.001) between the effects of COVID-19 on students' health status or their **relatives** among the students whose lives were negatively affected (*T* = 937) and the total PSS-10 and the total stress-related symptoms and its four (emotional, cognitive, physical, and behavioral) domains (Table 4).

**Table 4:** Effect of COVID-19 on university students.

**Effect of COVID-19 Pandemic on university students**	***F***	**%**
**The pandemic affected students'lives directly or indirectly**		
• Yes	937	63.9
• No	530	36.1
**Effects of COVID-19 on Students'health status or their relatives**		
Infected with mild COVID-19 symptoms	708	72.8
• Isolation at home for a period of time	842	57.4
• Isolation in hospital with more serious symptoms	74	7.6
• Admitted to ICU with severe symptoms	46	4.7
• Death of one of your relatives after infection	377	38.8
**Effects on the studying**		
• The exams had been postponed	664	45.3
• The study was online	783 239	53.4
• The exams were canceled and replaced with researches		30.2
**Correlation between the effects of COVID-19 on students' health status or their relatives among the students whose lives were negatively affected during the pandemic (*****T*** **= 937) and the following**	***R***	***P***
Emotional stress-related symptoms	0.73	0.00^*^
Physical stress-related symptoms	0.78	0.00^*^
Behavioral stress-related symptoms	0.81	0.00^*^
Cognitive stress-related symptoms	0.80	0.00^*^
The total stress-related symptoms	0.91	0.00^*^
The PSS total score	0.68	0.00^*^

* p <0.05 there was a statistical significant difference/r for the correlation coefficient.

### PSS-10 and its predictors in university students

The majority of the studied university students suffered from moderate and severe stress, 960 (65.4%) and 474 (32.3%), respectively. The mean ±SD) of the PSS was significantly (*p* < 0.05) higher among females (24.5 ± 5.7), non-Egyptians (32.2 ± 6.5), students with low GPA (25.1 ± 6.1), and students who suffered from both organic and psychological disorders (26.6 ± 4.9). On the other hand, it showed no statistically significant relationship (*p* > 0.05) with the type of study, residence, and relationship status. The total PSS score was significantly (*p* < 0.05) correlated with the coping strategies. The more the positive adapted coping strategies, the lower the PSS (*r* = −0.23), and positively correlated with the adapted negative coping strategies (Table 5).

**Table 5:** The Perceived Stress Scale (PSS-10) and its relationship with the demographic characteristics.

**The demographic characteristics**	**Total PSS score**	**Perceived stress scale (PSS-10) 24 (24.2** ±**5.9) (5–40)**			***P*-values of chi-square test**
		**Mild 34 (2.3%) *F* (%)**	**Moderate 960 (65.4%) *F* (%)**	**Sever 474 (32.3%) *F* (%)**	
**Age (y)**					
**Mean ±SD**		21.4 ± 1.6	21.6 ± 1.4	21.1 ± 1.9	0.98
**Sex**					
Female	24.5 ± 5.7	23 (67.6)	743 (77.4)	391 (82.5)	0.04^*^
Male	22.7 ± 5.9 *P* = 0.00^*^^##^	11 (32.4)	217 (22.6)	82 (17.5)	
**Nationality**					
Non-Egyptian	32.2 ± 6.5	2 (5.9)	23 (2.4)	10 (2.3)	0.04^*^
Egyptian	24.2 ± 5.9 *P* = 0.04^*^^##^	32 (94.1)	937 (97.6)	463 (97.7)	
**College**					
Practical	24.3 ± 5.9	26 (76.5)	692 (72.1)	354 (74.7)	0.47
Theoretical	23.9 ± 5.9 *P* = 0.11^##^	8 (23.5)	268 (27.9)	119 (25.3)	
**Emotional state**					
Single	24.2 ± 5.9	31 (91.2)	819 (85.3)	412 (86.9)	0.62
In a relationship	25.7 ± 6.2	1 (2.9)	33 (3.4)	20 (4.4)	
Engaged	24 ± 5.4	2 (5.9)	78 (8.1)	28 (5.9)	
Married	23.5 ± 5.5 *P* = 0.78^#^	0 (0.0)	30 (3.1)	13 (2.7)	
**Residence**					
Campus	24.2 ± 5.9	7 (20.6)	143 (14.9)	66 (13.9)	0.76
Urban	24.3 ± 5.9	14 (41.2)	424 (44.2)	215 (45.6)	
Rural	23.9 ± 5.8 *P* = 0.45^#^	7 (20.6)	393 (40.9)	192 (40.5)	
**GPA**					
Excellent (A)	22.9 ± 5.9	14 (41.2)	222 (23.1)	76 (16.0)	0.00^*^
Very good (B)	24.2 ± 5.6	7 (20.6)	397 (41.4)	189 (39.9)	
Good (C)	24.6 ± 5.9	11 (32.4)	256 (26.7)	150 (31.6)	
Fairly good (D)	25.1 ± 6.1 *P* = 0.00^*^^#^	2 (5.9)	85 (8.9)	58 (12.2)	
**Had chronic diseases**					
Non	22.6 ± 5.6	23 (67.6)	565 (58.9)	173 (36.5)	0.00^*^
Organic	22.2 ± 5.6	7 (20.6)	77 (8.0)	21 (4.4)	
Psychological	26.4 ± 5.9	2 (5.9)	161 (16.8)	133 (27.8)	
Both	26.6 ± 4.9 *P* = 0.00^*^^#^	2 (5.9)	157 (16.4)	148 (31.2)	
**The coping strategies**			R (p)		
Positive			−0.23 (0.00^*^)		
Negative			0.41 (0.00^*^)		

* p <0.05–there is a statistically significant relationship.

# p-values of F test.

## p-values of student t-test.

Females OR [95% C.I] 2.65 [1.61–11.5], lower GPA OR [95% C.I] 3.36 [1.5–14.9], those with psychological disorders OR [95% C.I] 4.58 [4.07–19.6], and those with both (organic and psychological disorders) OR [95% C.I] 4.79 [1.12–20.4] were factors (predictors) that increased the risk of being under moderate to severe stress (Table 6).

**Table 6:** Predictors of moderate to severe stress among university students.

	**OR [95% confidence Intervals C.I]**	***P***
**Sex**		
Male (reference)	**—**	
**Females**	2.65 [1.61–11.5]	0.019^*^
**Nationality**		
Non-Egyptian (reference)	—	
Egyptian	0.55 [0.27–1.14]	0.11
**GPA**		
Excellent (A) (reference)	—	
Very good (B)	2.51 [1.12–5.5]	0.03^*^
Good (C)	2.72 [1.09–6.8]	0.02^*^
Fairly good (D)	3.36 [1.5–14.9]	0.001^*^
**Had chronic diseases**		
Non (reference)	—	
Organic	0.44 [0.18–1.04]	0.06
Psychological	4.58 [4.07–19.6]	0.04^*^
Both	4.79 [1.12–20.4]	0.03^*^

* p <0.05–there is a statistically significant relationship.

### Coping strategies among the university students studied

**Figure 2:**
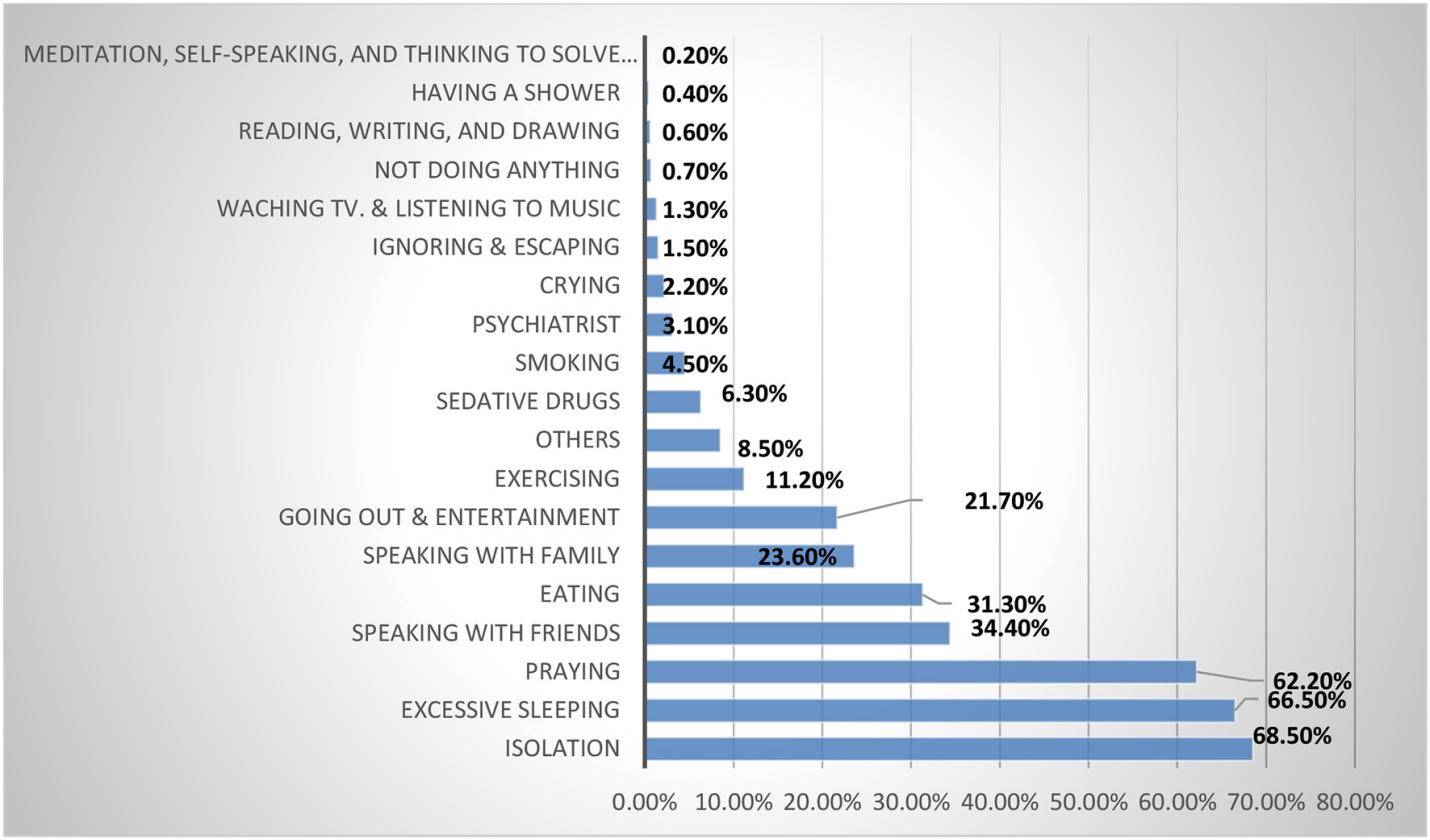
The frequency of the stress-relieving strategies among the studied university students.

As regards coping strategies during stressful times, the recruited university students 1,005 (68.5%) preferred isolation, 913 (66.5%) excessive sleeping, and praying (912, 62.2%). The least common strategies reported were 46 (3.1%) consulting a psychiatrist and 0.2% meditating (Figure 2).

## Discussion

During the third wave of the COVID-19 pandemic, the majority of university students in Egypt reported that the pandemic affected their lives in either direct or indirect ways. The majority suffered from moderate to severe perceived stress that was manifested by many physical, emotional, behavioral, and cognitive stress-related symptoms. Both students' perceived stress scales and stress-related symptoms had many determinants that we discussed in detail later.

### Physical symptoms of stress

**In terms of nonspecific stress-related symptoms**, we reported that 1212 (82.6%) of university students experienced stress-related fatigue, also known as “adrenal fatigue,” as revealed by (42–44). Stress impairs adrenal gland function, which has an impact on energy levels *via* 16 different mechanisms in the brain, gut, immune system, endocrine system, and mitochondria (the cellular energy generators of your body). e.g., It depletes the endocannabinoid system, changes the balance of neurotransmitters in the brain (serotonin, dopamine, and acetylcholine), disrupts the hypothalamus-pituitary-adrenal (HPA) axis, and reduces thyroid hormone levels (45). While 853 people (58.2%) reported stress-related dizziness, this was consistent with the stress effect on the vestibular system. This proved the causal association between stress and dizziness (46–48).

**As regards the stress-related neuromusculoskeletal system**, it is significantly impacted by stress, with low back pain accounting for 1,036 (70.6%), neck pain accounting for 994 (67.8%), and shoulders and arms pain accounting for 929 (63.3%) of university students. This is consistent with the findings of numerous studies conducted in a variety of workplaces in a variety of countries that demonstrated a link between stress and musculoskeletal pain complaints (49–52).

**In terms of stress-related neurological symptoms**, 879 (59.9%) of university students reported shakiness in their extremities, such as their hands. In conformity with the information provided in (53). When the body prepares to deal with a stressor, it perceives anxiety as a signal that there is a need to evacuate from danger, thus muscles become ready to respond, resulting in trembling, twitching, and even shaking (54). Additionally, stress can exacerbate psychogenic tremors [underlying psychiatric problems like depression or post-traumatic stress disorder (PTSD)], but these psychogenic tremors can be reduced or eliminated when people are preoccupied (53).

**Tension-type headaches (TTH)** and tightness around the head were reported by 1,225 (83.5%) of university students consistently with (55–57). Although the fundamental causes are currently unclear, Simultaneously, research in healthy humans is beginning to shed light on the link between stress and pain processing in the central nervous system, particularly in the central pain systems that are thought to be dysfunctional in TTH. Moreover, 861 (58.7%) of students suffered from **stress migraine** (a debilitating, throbbing, and severe pain present only on one side of the head) similar to (58, 59). Stress and anxiety can also trigger TTH and migraine and contribute to its chronicity (60). But stress headaches and migraine attacks mess with your head differently.

**In terms of stress-related dermatological symptoms**, 1,104 (75.3%) of university students experienced *hair loss during stressful situations*, making it the third most common symptom after headaches and chronic fatigue. According to (26), corticosterone (stress hormone) is a systemic inhibitor of hair follicle stem cell function. Chronic stress disrupts tissue homeostasis, resulting in hair loss. While 883 (60.2%) of those surveyed *had acne*, acne is strongly linked to stress, according to (27–29). Acne in stressed females may be due to associated menstrual disturbances, which 667 female university students (61–65) reported having.

**Ophthalmology stress-related symptoms** were reported by 889 (60%) students in the form of blurred vision, double vision, fogginess, and any other eye-related complaints. Consistently with (30), who found that symptomatic vitreous floater patients showed substantial levels of psychological distress and that the severity of floater symptoms was significantly associated with that distress. They agreed with (31) that dry eye disease is more common in those who are depressed, anxious, or stressed.

**As regards stress-related respiratory symptoms**, Similar to (66, 67), in this study we reported shortness of breath among 668 (45.5%) and rapid breathing among 581 (39.6%) university students, known as ***psychogenic or anxiety dyspnea***, or dyspnea catastrophizing (dyspnea that is not related to effort). These difficulties in filling the lung are correlated with the anterior cingulate cortex (ACC) in the brain. The ACC's role is to process negative affective states such as anxiety (68–70).

**Stress-related cardiovascular diseases (CVD)** were palpitations of 866 (59%), chest pain of 532 (36.3%), and increased blood pressure of 424 (28.9%), respectively. Musey et al. (71, 72), Redina and Markel (73), Damtie et al. (74) found similar results. Because the sympathetic nervous system and the hypothalamus-pituitary-adrenal (HPA) axis are engaged, more catecholamines, glucocorticoids, and inflammatory cytokines are produced, resulting in increased cardiac output, skeletal muscle blood flow, salt retention, and cutaneous vasoconstriction (75).

**Stress-related gastrointestinal (GIT) symptoms** included bloating sensation and abdominal discomfort as the most reported symptoms by 861 (58.7%), and abdominal pain was reported by 822 (56%). According to prior research (76–79), negative emotional stress both increases and exacerbates IBD symptoms. Catecholamines and cortisol are both released by the brain in stressful situations, and the digestive system can't get away from the combined effects of both hormones on the body, which reduce intestinal motility (80).

In agreement with (61–65), we found that 667 (57.6%) of female participants had **menstrual disturbances**, such as irregular and/or heavy menses. This is due to stress's role in lowering circulating gonadotropins and gonadal steroid hormones; consequently, persistent stress can result in full reproductive function impairment (80).

### Negative cognitive symptoms associated with stress

The main complaints were anxiety and racing thoughts, followed by poor concentration, memory issues, poor judgment, and only perceiving the negative. It was in line with (81–84). Stress can shut down many centers in the prefrontal cortex, which orchestrates the brain's activity for intelligent regulation of behavior, thought, and emotion and allows the primitive brain to take over, causing mental paralysis and panic. In addition to losing self-control, they are likely to make poor lifestyle choices, including bad decisions (85).

### In terms of stress-related emotional symptoms

Moodiness, irritability, or rage are the most commonly reported symptoms, followed by depression and unhappiness, loneliness and isolation, anxiety, and agitation. These findings are in accordance with others (86–89). This is the body's hormonal stress-response mechanism, whereby stress activates the hypothalamic-pituitary-adrenal (HPA) axis, and glucocorticoids, including the cortisol hormone, are released into the bloodstream, increasing the risk of depression. Stress also decreases the volume of the hippocampus (the brain part that affects emotional reactions). This could result in depressive symptoms (90).

### In terms of stress-related behavioral symptoms

Excessive sleeping, withdrawing from gatherings, and preferring isolation were the most commonly reported symptoms, respectively. While smoking was the least reported behavioral change. These findings are consistent with outcomes from previous studies (91–94).

Seven hundred twenty-five (49.54%) students reported an increase in appetite, while 854 reported a reduction (58.2%). This depends on the type of stress, because stress alters food intake, raising or decreasing it (95, 96). In the short term, stress can reduce appetite as the nervous system sends signals to the adrenal glands to secrete epinephrine, which triggers the body's fight-or-flight response that temporarily puts eating on hold. But if stress persists, the adrenal glands release cortisol, which increases appetite and increases the motivation to eat (97).

Stress activates the sympatho-adreno-medullary (SAM) and HPA systems. Hormones like melatonin and others from the HPA axis modulate the sleep-wake cycle, while its dysfunction can disrupt sleep (98).

### Effect of the COVID-19 pandemic on university students' lives

We found that 937 (63.9%) reported that the COVID-19 pandemic badly affected their lives, either directly or indirectly. Consistently with (99) which proved that COVID-19 confinement directly caused moderate to severe stress among university students. And agreeing with (76), stated that COVID-19 anxiety was significantly related to Crohn's disease symptom severity and social dysfunction. There were many reported causes of this stress, including infection with COVID-19, which was reported by a total of 828 students with different severities. Three hundred and seventy-seven (38.8%) of students experienced a relative's death with COVID-19, and 842 (57.4%) experienced home isolation. This was because on May 31st, the total number of confirmed cases was 267,171 cases, and 15,309 deaths by default affected all inhabitants in Egypt, including university students (100), and stress related to the high level of widespread vaccine hesitancy and worries about the COVID-19 vaccine, that only around one million citizens have been vaccinated out more than 100 million (101). In addition to the effect of the pandemic on their study, 783 (53.4%) studied online, 664 (45.3%) delayed exams, and 239 (30.2%) replaced. Even though Egypt thought these strategies would help stop the spread of the pandemic, all of these new experiences in learning and exams could be stressful and difficult for both the universities and students at this time (102). Furthermore, there are other factors like family responsibilities, personal health concerns, health problems that affected the family, and the economy that played a role.

In the students whose lives were adversely affected (*T* = 937) during the pandemic, there was a moderate to good (*r* > 0.76), positive or direct statistically significant association (*p* = 0.001) between the effects of COVID-19 on students' health status or that of their relatives, and the overall stress-related symptoms and its four subtypes. In addition to COVID-19-related stress, the post-COVID-19 condition may be to blame for this. Additionally, as many as 828 (88.4%) of the population may have post-COVID-19 syndrome. Although there are many different, changing, and fluctuating clinical symptoms, fatigue and neurocognitive impairments are the most common. Post-COVID-19 syndrome, which manifests as a variety of emotional symptoms like depression and anxiety; physical symptoms like headaches, fatigue, and menstrual changes; behavioral symptoms like appetite and sleep disturbance; and post-COVID-19 cognitive impairment. Although post-COVID-19 syndrome is not well understood, its diagnostic criteria have not undergone adequate psychometric evaluation (103, 104). It is critical to distinguish it from stress-related symptoms.

### PSS-10, and its determinants

In May 2021, our study showed that the frequency of moderate to severe stress among university students was more than 97% (65.4% moderate and 32.3% severe). This can be attributed to a variety of factors, including the fact that 937 (63.9%) of them reported that the COVID-19 pandemic had a negative impact on their lives, either directly or indirectly. Furthermore, during stressful times, the majority of recruited university students adopted negative coping strategies, preferring isolation and excessive sleeping. Positive coping strategies, consulting a psychiatrist, and meditation were the least reported.

The frequency of moderate-to-severe stress varied in different years and countries, but all were lower than ours. For example, 92.5% in Pakistan in 2013, 88.9% in Minia, Egypt in April 2017, 71.9% in Saudi Arabia in 2012, 62.3% in Fayoum, Egypt in 2017, and 54% in Ain Shams University in 2016 (38). This may be attributed to the challenges of the COVID-19 pandemic because more than 78.0% of the recruited students were females, who are more susceptible to stress than males, and 377 (38.8) of them experienced the death of a family member as a result of COVID-19 (death of a loved one is the number two of the top five stress drivers).

**In terms of gender**, many studies (16, 105, 106) found a significantly higher PSS in females, consistent with our finding; OR [95% C.I] was 2.65 [1.61–11.5] (*p* = 0.00). Numerous neurobiological and behavioral factors may contribute to this gender disparity in stress response. Acute HPA, ACTH, and autonomic response levels in adult women have been found to be lower. Additionally, women's sexual hormones lower HPA and sympathoadrenal reactivity. As a result, the brain receives slow cortisol feedback, has a harder time controlling its stress response, and is more likely to experience depression in women. They are also vulnerable to the depressive impacts of interpersonal problems. When women engage in “tend-and-befriend” behaviors, their right prefrontal brain activity, cortisol levels, and left orbitofrontal cortex activity are all reduced. It involves lowering cortisol levels, the stress response, and attachment-related caregiving mechanisms that usually control sympathetic and HPA arousal, as well as the limbic system, which is made up of the ventral striatum, putamen, insula, and cingulate cortex (107).

Other research (108) showed no discernible difference in stress levels between men and women. Others claimed that male students had higher stress levels than female students. However, this might be due to the cultural, religious, and female obligations of certain groups. Cultural factors including relationship issues with parents and instructors (such as unfairness, mistreatment, and severe criticism) and substance abuse—most commonly using mild tranquilizers—were noted more frequently by Egyptian women. Mule and Barthel (16) addressed the societal changes in Egypt, where there has been an increase in women's engagement in the labor market and, to some extent, in politics. Additionally, globalization and exposure to Western culture have steered this traditionally Islamic nation toward alternate gender values. The lack of gender differences may also be attributed to the student body's extremely selectivity and homogeneity as well as its particular personal attributes that are valued in the competitive environment of medical school (17). In addition to their regular tasks, which include taking care of the home.

**In terms of nationality**, Non-Egyptians had significantly (*p* = 0.04) higher levels of stress (moderate to severe) than Egyptians, and being Egyptian was a protective factor OR 0.55, and 95% C.I [0.27–1.14], consistent with previous research that found international students to be more stressed than domestic students, which is described as acculturative stress, which is attributed mainly to the unfamiliar cultural and environmental changes, the different educational systems, and limited language proficiency (109, 110).

**In terms of academic performance**, students with low GPA were significantly more stressed than the others OR 3.36, and 95% C.I [1.5–14.9], (*p* = 0.00), which is in line with the results of other studies that have found that students with high academic performance experience less stress (105, 106). Similar studies have demonstrated that clinical outcomes like suicidality are influenced by academic stress. Subjects who are at risk for poor clinical outcomes frequently approach suicide by searching online for news and information about self-harm and suicidal behaviors, even if they are good ethical leaders (111). Additionally, affective temperament-types were more strongly and independently associated with poor clinical outcomes than were major depressive disorder diagnoses or other variables that might be linked to academic stress and poor clinical outcomes (112).

**In terms of comorbidity**, we found that university students with psychological disorders OR 4.58, 95% C.I [4.07–19.6] and those with both organic and psychological disorders OR 4.79, 95% C.I [1.12–20.4] had a significantly higher prevalence of stress (*p* = 0.00). That was also related to vaccination hesitancy as vaccination priority was given to patients with chronic diseases. In addition to the higher perceived severity and susceptibility (101).

**In terms of coping strategies, the** total PSS score was significantly (*p* < 0.05) correlated with the coping strategies. The more the positive adapted coping strategies, the lower the PSS (*r* = −0.23) and it was positively correlated with the adapted negative coping strategies. This explains why (we advise reading Doing What Matters in Times of Stress: An Illustrated Guide). The need to give people useful tools to help them manage stress is emphasized in the stress management book Coping with Distress. Self-help techniques can be practiced every day for as little as a few minutes (113). Consequently, it is crucial to create and implement health education programmes for college students.

### Stress coping strategies

According to the various coping techniques described by (40, 41). Coping strategies for stress are different from one another. Students resorted to the following ways to relieve stress: One thousand and five (68.5%) preferred isolation, while 913 (66.5%) preferred excessive sleeping as the most common coping method, although these coping strategies may be symptoms of stress (23). That is in line with the fact that, when things are stressful, students tend to avoid social situations by hiding or sleeping more than usual.

More than four hundred university students (31.3%) try eating as a way to emit the angry or stressful energy contained within. This can be attributed to the fact that stress increases appetite and a hunger sensation, in addition to sugar cravings, which serve as a symptom as well as a coping mechanism. Cravings lead to poor eating habits, such as eating highly processed foods that are low in protein and fiber but high in glycemic load. This causes inflammation, which suppresses the neurotransmitter orexin directly (which is key in regulating wakefulness and energy levels). Energy drinks, ironically, deplete your energy levels the most. Clinical studies also revealed high food consumption, specifically of palatable foods, during periods of psychological stress (95). Glucocorticoids make people eat more food that they find tasty. This is linked to reward-based eating as a way to lessen the stress response (92, 96).

Referring to the religious background of the Egyptian society, 912 (62.2%) university students were praying to relieve their stress. Five hundred four (34.4%) preferred speaking with a friend over talking with family. Three hundred forty-six (23.6%), because speaking with others can trigger hormones that relieve stress (23). Logically, students would share similar stressors based on their age and study nature. Therefore, they would understand each other more than their families. Only 11.02% adapted exercise that serve as a distraction from negative thoughts and pause the negative cycle of stress (114).

While the least followed coping method was smoking, 66 (4.5%), this is attributed to the fact that some people smoke as self-medication to ease feelings of stress. As nicotine creates an immediate and temporary sense of relaxation, people falsely think that smoking reduces stress and anxiety. But actually, smoking increases anxiety and tension, plus there its negative effects on health (115). This small percentage may be attributed to the fact that 78.9% of the recruited students were females, and smoking is not acceptable among females in Egypt due to cultural factors besides religious factors.

Coping strategies employed by students in dealing with academic stress vary among different studies. For example, in Nigeria, for example, the top six coping strategies were as follows: adopt a positive attitude; yell and scream; exercise; talk to friends; think about their goals to put things in perspective; and go shopping or to a place of worship (114). Students at the UEW Winneba Campus preferred the emotion-focused style to the problem-focused style in dealing with stress (116). A survey of undergraduate pharmacy students at the University of Khartoum revealed that the most frequent strategies were praying (84.4%) and maintaining some control over the situation (61.9%) (117). In Hong Kong, Chinese university students seemed to prefer positive coping strategies (namely, “Problem Solving” and “Seek Social Support”), especially “Problem Solving” (118), while in Poland, there are various coping strategies which are preferred by different categories of students (119).

Unfortunately, only 46 (3.1%) went to a psychiatrist, which helped to personalize the best healthy strategies to deal with stress according to everyone's needs and lifestyle (34). That may be attributed to the lack of students' awareness of their mental health, fearing stigma, the cost, and their tight timetable. There were also other ways with a minimal percent described by students. Only 3 (0.2%) of the recruited university students said they adopted relaxation techniques such as meditation, yoga, and deep breathing to reduce stress and boost feelings of joy through activation of the body's relaxation response, which increases concentration, reduces stress and anxiety, alleviates depression, and enhances our overall mental health (23).

Lastly, stress, according to our research, manifests itself not just as psychological distress but also as a variety of physical, cognitive, emotional, and behavioral manifestations. Moank of factors that affected by stress, appeared to increase during covid (120–122).

## Strength

The study was conducted on a large sample. The sample was randomly stratified to represent all the university students in Egypt. It included students from 20 governorates, 30 public and private universities, 13 practical and 13 theoretical colleges, and from all the academic grades.

The questionnaire used to get the data was well-structured, validated, and detailed. It asked 54 questions about stress-related symptoms and how often people said they had them, on a scale of one to four (never, rarely, often, sometimes, and always).

The study didn't just look at the common psychological stress-related symptoms. It also looked at the physical, cognitive, emotional, and behavioral aspects of stress, which haven't been talked about as much in the literature before.

Data was collected at the end of the semester in May 2021, during the 3^rd^ wave of the COVID-19 pandemic. Students are typically stressed during this time as they prepare for finals, which gives an idea of the current associated symptoms. This decreases the recall bias in this cross-section study.

## Limitations

First, our study has all the limitations of observational studies, such as bias and confounding issues. Second, the study depended on an online self-administered questionnaire, which reduced the accuracy of collected data because of recall and availability bias. Furthermore, it was a self-administered questionnaire without a complete clinical examination to exclude other organic causes. Third, the poor response rate from some groups, such as males and theoretical college students. Fourth, when we talk about new types of stress that haven't been studied before, it's hard to tell which of the findings about stress are caused by the end of the semester or COVID problems. Also, the students used a variety of coping techniques to deal with their stress, which we don't know about. Fifth, we couldn't prove the associations as a cross-sectional study. Finally, there is no baseline or pre-pandemic assessment.

## Conclusion

More than half of the recruited students reported that the COVID-19 pandemic badly affected their lives, either directly or indirectly. Perceived stress and stress-related symptoms were prevalent among university students during the third wave of the COVID-19 pandemic, which was manifested with many physical, cognitive, emotional, and behavioral stress-related symptoms. The top 10 prevalent physical symptoms were headaches, chronic fatigue, hair loss, low back pain, neck pain, shoulders and arm pain, ophthalmological symptoms, acne, shakiness of extremities, and palpitations, respectively. The most reported symptoms regarding the cognitive, emotional, and behavioral aspects were anxiety and racing thoughts, moodiness, and excessive sleeping, respectively. Perceived stress is significantly higher among females, international students, those with low GPA, and students who have organic and psychological diseases. The majority of recruited university students during stressful times adopted negative coping strategies (preference for isolation and excessive sleep), while positive coping strategies (consulting a psychiatrist and meditation) were the least reported.

## Recommendations

(1) In order to raise awareness of students' mental health, we propose that the results of this study, which demonstrate how demanding academic life can be, be communicated with stakeholders at every university. Ours emphasizes the stresses of academic life, calling for increased attention to and support for students' mental health while also urging universities to provide funded psychiatric visits and other stress-reduction measures.

(2) Developing and implementing health education programs for college students, particularly risky groups (females, international students, students with low GPA, and students with organic and psychological diseases), about the various aspects of stress that can affect them in order to avoid unnecessary investigations; reducing student drug abuse because they frequently use over-the-counter drugs to relieve pain or other unexplained physical symptoms; and early detection with a better diagnosis. Furthermore, educating students on the physical symptoms of stress will reduce the number of times they visit unnecessary specialists to explain the effects of academic stress on their health; increase awareness of stress-related symptom, and offer the best coping mechanisms in order to improve their mental health and well-being.

(3) Providing affordable, accessible, and confidential mental health support services in all colleges and universities through well-trained social workers, life coaches, and psychiatrists for the rapid and effective management of all related stress and mental and psychological disorders among university students.

(4) Governments and the media should try to get rid of the old-fashioned stigma of mental disorders and make it more common for people to go to a psychiatrist.

(5) (Doing What Matters in Times of Stress) is based on informed evidence and extensive field testing for anyone who experiences stress, wherever they live and whatever their circumstances. An Illustrated Guide that we recommend: Coping with Distress is a stress management book for dealing with adversity. The goal of the handbook is to provide people with practical tools to assist them in managing stress. Self-help approaches can be practiced in as little as a few minutes per day. The guide can be used alone or in conjunction with the audio exercises, which are available in 17 languages (113).

(6) Additional research would be conducted on different populations in different countries to prove and strengthen these findings, to focus on each reported symptom, to try to clarify the mechanisms of these associations because many symptoms occur with unknown mechanisms; to improve diagnosis with early detection and proper management through referral to a psychiatrist; to study and compare the effectiveness of each coping strategy, and to provide recommendations for people.

## Data availability statement

The datasets used and/or analyzed during the current study are available from the corresponding author upon request.

## Ethics statement

The studies involving human participants were reviewed and approved by Institutional Review Board at Zagazig University (#6925). The patients/participants provided their written informed consent to participate in this study.

## Author contributions

Conceptualization: MA and SA. Methodology: MA and SA. Software: JS. Supervision: MA, SA, and JS. Validation: MA, MR, and SA. Formal analysis and supervision: SA. Data curation: MA, FI, ME, MK, MR, and SA. Writing—review and editing and visualization: MA and SA. All authors writing—original draft preparation and have read and agreed to the published version of the manuscript.

## Conflict of interest

The authors declare that the research was conducted in the absence of any commercial or financial relationships that could be construed as a potential conflict of interest.

## Publisher's note

All claims expressed in this article are solely those of the authors and do not necessarily represent those of their affiliated organizations, or those of the publisher, the editors and the reviewers. Any product that may be evaluated in this article, or claim that may be made by its manufacturer, is not guaranteed or endorsed by the publisher.

## Abbreviations

Q: Questions
F: Frequency
%: Percentage
SD: Standard deviation
PSS: Perceived Stress Scale
fig: Figures
ANOVA: Analysis of Variance
HPA: Hypothalamus-pituitary-adrenal axis
PTSD: Post-Traumatic Stress Disorder
TTH: Tension-Type Headaches
ACC: Anterior cingulate cortex
CVD: Cardiovascular Diseases
GIT: Gastrointestinal Tract
SAM: Sympatho-Adreno-Medullary.
