# Supplementary material for: Cognitive, emotional, physical, and behavioral stress-related symptoms and coping strategies among university students during the third wave of COVID-19 pandemic
Source: Front Psychiatry. 2022 Sep 16;13:933981. doi: 10.3389/fpsyt.2022.933981 (PMC9523087; doi:10.3389/fpsyt.2022.933981)
Supplement: Supplementary file 1 [file Data_Sheet_1.docx]

**Supplementary materials**

**Supplementary material 1.** *Participants’ governorates and universities*

| Governorates | University | Frequency | Percent (%) |
| --- | --- | --- | --- |
| El Sharkia | Zagazig | 829 | 56.5 |
| Cairo | Al-Azhar | 29 | 2.0 |
| Cairo | Ain Shams | 44 | 3.0 |
| Cairo | BUC | 21 | 1.4 |
| Cairo | 6 October | 24 | 1.6 |
| Cairo | Helwan | 28 | 1.9 |
| Cairo | MUST | 9 | .6 |
| Cairo | BUE | 3 | .2 |
| Cairo | ERU | 4 | .3 |
| Cairo | GUC | 1 | .1 |
| Cairo | Heliopolis | 47 | 3.2 |
| Al Giza | Cairo | 45 | 3.1 |
| Al Qalyubia | Benha | 35 | 2.4 |
| Al Dakahlia | Mansoura | 33 | 2.2 |
| Al Ismailia | Suez Canal | 32 | 2.2 |
| Al Monofeya | Monofeya | 26 | 1.8 |
| Alexandria | Alexandria | 26 | 1.8 |
| Sohag | Sohag | 19 | 1.3 |
| Beni suef | Beni suef | 15 | 1.0 |
| Al Gharbia | Tanta | 15 | 1.0 |
| Al Minya | Al Minya | 14 | 1.0 |
| Assiut | Assiut | 14 | 1.0 |
| Kafr El Sheikh | Kafr El Sheikh | 13 | .9 |
| Al Fayoum | Al Fayoum | 11 | .7 |
| Port said | Port said | 8 | .5 |
| Aswan | Aswan | 6 | .4 |
| Damietta | Damietta | 3 | .2 |
| Suez | Suez | 3 | .2 |
| Marsy Matrouh | Matrouh | 1 | .1 |
|  | Others | 109 | 7.4 |
| Total |  | 1467 | 100 |

**Supplementary material 2.** *Practical and theoretical colleges that participants belonged to*

| Type | College | Frequency | Percent  % |
| --- | --- | --- | --- |
| Practical | Medicine  Dentistry  Physical Therapy  Pharmacy  Veterinary medicine  Science  Applied science  Applied arts  Engineering  Bio technology  Nursing  Computer Science  Agriculture | 503  31  20  187  28  44  2  65  63  1  16  18  18 | 34.3  2.1  1.4  12.7  1.9  3  .1  4.4  4.3  .1  1.1  1.2  1.2 |
| Total | 13 faculties | 996 | 67.8 |
| Theoretical | Arts  Archaeology  Al-Alson  Press and Media  Home Economy  Commerce  Education  Specific Education  Physical Education  Law  Social Science  Kindergarten Education  Languages and Translation | 145  8  27  1  2  85  61  8  6  80  8  9  1 | 9.9  .5  1.8  .1  .1  5.8  4.2  .5  .4  5.5  .5  .6  .1 |
| Total | 13 faculties | 441 | 30 |
| Others |  | 30 | 2 |
| Total |  | 1467 | 100 |

**Supplementary material 3.** *Questionnaire used to collect the data in both Arabic and English*

| **Questionnaire in English** | **الاستبيان باللغة العربية** |
| --- | --- |
| **Section 1** | **القسم الاول** |
| **Sex**  Male  female | **الجنس**  ذكر  أنثى |
| **Age** | **العمر بالسنين** |
| **Nationality**  Egyptian  Non-Egyptian | **الجنسية**  مصري  غيرمصري |
| \| **University name**  Aswan \| \| --- \| \| Assiut \| \| Al-Azhar \| \| Alexandria \| \| GUC \| \| BUE \| \| ERU \| \| Zagazig \| \| Suez \| \| Al fayoum \| \| Cairo \| \| Mansoura \| \| Monofya \| \| Minya \| \| BUC \| \| Benha \| \| Beni seuf \| \| Port said \| \| 6 October \| \| Helwan \| \| Damietta \| \| Sohag \| \| Tanta \| \| Ain Shams \| \| Suez Canal \| \| Kafr El Sheik  Matrouh  Al-Arish  AL-Aqsor \| \| MUST  AUC  Al-Nile  Heliopolis  Others \| | **إسم الجامعة**  الزقازيق  القاهرة  عين شمس  بنها  المنصورة  الإسكندرية  الفيوم  حلوان  كفر الشيخ  المنوفية  طنطا  بورسعيد  الأزهر  دمياط  قناة السويس  السويس  أسيوط  سوهاج  المنيا  بني سويف  مطروح  الأقصر  أسوان  العريش  السادس من أكتوبر  مصر للعلوم و التكنولوجيا  هيليوبوليس  بدر  النيل  الروسية  البريطانية  الأمريكية  الألمانية  أخرى |
| **Type of study**  Theoretical  Practical | **الدراسة بالكلية**  كلية نظرية  كلية عملية |
| **College**  Medicine  Dentistry  Physical Therapy  Pharmacy  Veterinary medicine  Science  Applied science  Applied arts  Engineering  Bio technology  Nursing  Computer Science  Agriculture  Arts  Archaeology  Al-Alsun  Press and Media  Home Economics  Commerce  Education  Specific Education  Physical Education  Law  Social Science  Kindergarten Education  Languages and Translation  Others | **الكلية**  الطب البشري  الأسنان  الصيدلة  العلاج الطبيعي  الطب البيطري  العلوم  الهندسة  التربية  الآداب  الحقوق  التجارة  الزراعة  الحاسبات والمعلومات  التكنولوجيا والتنمية  التربية الرياضية  التمريض  التربية النوعية  العلوم الاجتماعية و الخدمة الاجتماعية  الإعلام  الفلسفة و المنطق  الآثار  الفنون التطبيقية  الفنون الجميلة  الألسن  سياسة و اقتصاد  لغات و ترجمة  الاقتصاد المنزلي  رياض أطفال  العلوم التطبيقية  سياحة و فنادق  بيوتكنولوجي  أي كلية أخرى |
| **Academic level**  First year  Second year  Third year  Fourth year  Fifth year  Sixth year  Internship year | **الفرقة**  الاولى  الثانية  الثالثة  الرابعة  الخامسة  السادسة  طبيب امتياز |
| **Marital status**  Single  Engaged  In relationship  Married | ا**لحالة الاجتماعية**  أعزب  مخطوب\ة  في علاقة  متزوج\ة |

| **Residence**  Rural  Urban with family  Urban beside university (Campus) | **مكان السكن**  ريف  حضر مع الأسرة  حضر في سكن الجامعة |
| --- | --- |
| **Average academic degree during the past college years (GPA)**  Excellent (A)  Very good (B)  Good (C)  Fairly good (D) | **ما هو متوسط تقديرك خلال سنوات الكلية**  امتياز  جيدجداً  جيد  مقبول |
| **Do you suffer from any of this health problems?**  Psychological diseases  Organic disease  Both  Non | **هل تعانى من أيٍ من المشاكل الصحية التالية**  أمراض أو اضطرابات نفسية  أمراض أو اضطرابات عضوية  كلاهما  لا أعانى من أية أمراض |
| **If you suffer from any physical or psychological disease, please mention it?** | **إذا كنت تعاني من أى أمراض نفسية أو عضوية، أُذكرها** |
| **Section 2** | **القسم الثاني** |
| **Have you ever suffered from any of these physical symptoms during the stressful periods and without a clear medical cause?**  Low back pain  Neck pain  Shoulders and arms pain  Clenching  Dizziness  Headache  Migraine  Chronic fatigue  Increased body temperature  Hotness or redness in face, ears, neck or chest (Hot flashes)  Increased sweating  Slurred speech  Dry mouth  Painful oral ulcers  Difficulty swallowing  Heartburn or regurgitation after eating  Vomiting  Stomach ache after eating  Abdominal pain  Bloating sensation and abdominal discomfort  Diarrhea  Constipation  Increased weight or obesity  Decreased weight  Shakiness of your extremities such as hands  Frequent Cold or Flu  Shortness of breath  Increased breathing  Chest pain  Palpitations  Increased blood pressure  Acne or any other skin problems  Noticeable hair loss  Frequent itching in any part of the body  Menstrual disturbance  Eye problems such as blurred vision, double vision, Fogginess, or any other problems | **هل سبق أن عانيت من أي من الأعراض الجسدية الآتية خلال فترة الضغوط النفسية وبدون سبب طبي واضح**  آلام في أسفل الظهر  آلام الرقبة  آلام في الكتفين و الذراعين  شدة الجزعلي أسنانك  الشعور بالدوار الصداع  الصداع النصفي  الإجهاد العام أو الإرهاق الشديد بشكل مزمن  ارتفاع درجة حرارتك  احمرار أو سخونة بالوجه أوالأذن أوالرقبة أوالصدر  زيادة التعرق  التلعثم بالكلام أو صعوبة في الكلام  جفاف الفم  وجود قرح مؤلمة بالفم  صعوبة في البلع  الإحساس بحرقان في منطقة الصدرأو ارتجاع الطعام بعد الاكل  الرغبة في التقيؤ أو التقيؤ بالفعل  آلام في المعدة خاصةً بعد الأكل  آلام في البطن  الإحساس بالانتفاخ وعدم الراحة  الإسهال  الإمساك  زيادة الوزن أو السمنة  نقصان الوزن  الشعور باهتزاز أو ارتعاش في أيٍ من أطرافك كاليدين مثلاً  نزلات برد أو إنفلوانزا متكررة  صعوبة أو ضيق في التنفس  زيادة في سرعة تنفسك  آلام في الصدر  زيادة في ضربات القلب  ارتفاع ضغط الدم بشكل ملحوظ  حَب الشباب أو أي مشاكل أخرى بالبشرة  تساقط الشعر بشكل ملحوظ  الحكة أوالهرش في أي مكان بالجسم بشكل متكرر  اضطراب وعدم انتظام أوغزارة بالدورة الشهرية على غير المعتاد  مشاكل في العين كالزغللة أوالرؤية المزدوجة أو الضبابية أو أي مشاكل اخرى |
| **Have you ever suffered from any of these mental or cognitive symptoms during stressful periods?**  Poor concentration  Memory problems  Anxiety or Racing thoughts  Seeing only the negative  Inability to take proper decisions (poor judgment | **هل سبق أن عانيت من أيٍ من الأعراض الفكرية أوالعقلية التالية خلال فترات الضغوط النفسية**  عدم القدرة على التركيز  عدم القدرة على التذكر جيداً و النسيان  الإحساس المستمر بالقلق والتوتر أوالتفكير الزائد  الرؤية السلبية لكل شيئ  عدم القدرة على إتخاذ القرار السليم |
| **Have you ever suffered from any of these psychological or emotional symptoms during stressful periods?**  Depression and unhappiness  Anxiety and agitation  Feeling of loneliness and isolation  Moodiness, irritability or anger  Other psychological or emotional health problems | **هل سبق أن عانيت من أيٍ من الأعراض النفسية التالية خلال فترات الضغوط النفسية**  الاكتئاب و الإحساس بعدم السعادة  التهيج العصبي و التوتر  الشعور بالوحدة و الانعزال  تقلب المزاج أو التهيج أو الشعور بالغضب  أي مشاكل نفسية أخرى |
| **Have you ever suffered from any of these behavioral symptoms during stressful periods?**  Increased appetite  Decreased appetite  Excessive sleeping  Insomnia  Withdrawing from gatherings and preferring isolation  Neglecting duties and responsibilities  Smoking to relieve stress  Nail biting to relieve stress | **هل سبق أن عانيت من أيٍ من الأعراض السلوكية التالية خلال** **فترات الضغوط النفسية**  زيادة الشهية  نقص الشهية  النوم لفترات طويلة  عدم القدرة على النوم و الأرق  تفضيل الانسحاب من التجمعات و حب العزلة  إهمال واجباتك ومسئولياتك المعتادة  التدخين للتخفيف من التوتر  عض أظافرك لمحاولة التخفيف من التوتر |

| **Section 3** | **القسم الثالث** |
| --- | --- |
| **Did COVID-19 Pandemic affect your life directly or indirectly?**  Yes  No | **هل أثر فيرس كورونا المستجد عليك بشكل مباشر أو غير** **مباشر**  نعم  لا |
| **If your answer was Yes, select what you or one of your relatives suffered from during the pandemic? (You can choose more than one answer)**  Got COVID-19 with mild symptoms and isolated at home only  Isolation in hospital with more severe symptoms  Admitted to ICU with serious symptoms  Death of one of your relatives after infection | إ**ذا كانت إجابتك بنعم , اختر ما عانيت منه أنت أو أحد أقاربك خلال الوباء (يمكن اختيار أكثر من إجابة)**  الإصابة بالفيرس بأعراض خفيفة واكتفيت بالعزل المنزلي فقط  العزل في المستشفى وبأعراض أكثر حدة  دخول العناية المركزة بأعراض خطيرة  وفاة أحد أقاربك بعد إصابته |
| **Choose from the following what you have passed through during the pandemic and negatively affected your mental health?**  Your exams have been delayed  The study was online  Your exams were cancelled and replaced with researches  Isolation at home for a period of time | **اختر من الآتي ما مررت بيه خلال فترة الوباء وأدى للتأثير عليك نفسياً**  تم تأجيل امتحاناتك  تم إلغاء امتحاناتك و استبدالها بأبحاث علمية  تمت الدراسة عن بُعد (أونلاين)  انعزلت في منزلك لفترة من الوقت |
| **Section 4** | **القسم الرابع** |
| **During the last month, how often have you been or felt or passed through any of the following?**   - upset because of something that happened unexpectedly? - you were unable to control the important things in your life? - nervous and stressed? - confident about your ability to handle your personal problems? - things were going your way? - you could not cope with all the things that you had to do? - you been able to control irritations in your life? - you were on top of things? - you been angered because of things that happened that were outside of your control? - you felt difficulties were piling up so high that you could not overcome them? | **خلال الشهر الماضي, كم مرة تعرضت أو أحسست أو شعرت بأياً من الآتى؟**   - أزعجك حدوث شيء غير متوقع - أنك لا تستطيع التحكم في الأمور المهمة في حياتك - التوتراوالعصبية - الثقة في قدرتك على ادارة مشاكلك الشخصية - أن الأمورتسير لصالحك - أنك لا تستطيع القيام بكل الأشياء التي كان عليك أن تقوم بها - استطعت السيطرة على الأمورالمزعجة (المثيرة) في حياتك - أنك مسيطر على الأمور - غضبت بسبب وقوع أشياء خارجة عن إرادتك - أن المصاعب تتراكم لدرجة لا تستطيع التغلب عليها |
| **Section 5** | **القسم الخامس** |
| **How do you deal or handle stress usually? (You can select more than answer)**   - Isolation - Exercise - Sleeping too much - Speaking with family - Speaking with friends - Getting out and entertainment - Eating - Praying - Sedative drugs - Smoking - Psychiatrist - Others | **كيف تتعامل مع التوتر والضغط النفسي غالباً (يمكنك اختيار أكثر من إجابة)**   - التحدث مع أحد أفراد عائلتك - التحدث مع أصدقائك - الخروج والترفيه - الانعزال - تناول أدوية مهدئة - التدخين - ممارسة الرياضة - النوم بكثرة - الأكل - الصلاة والدعاء - الذهاب لطبيب نفسي - أخرى |
| **If you chose others, please mention it.** | إ**ذا اخترت أخرى، أذكر طريقتك في التعامل مع الضغط النفسي** |
